# Modeling and Simulating Dynamics of Complete- and Poor-Response Chronic Hepatitis B Chinese Patients for Adefovir and Traditional Chinese Medicine Plus Adefovir Therapy

**DOI:** 10.1155/2013/767290

**Published:** 2013-11-06

**Authors:** Lequan Min, Xiao Chen, Yongan Ye, Qun Zhang, Shuying Ru, Xiaoke Li

**Affiliations:** ^1^School of Mathematics and Physics, University of Science and Technology Beijing, Beijing 100083, China; ^2^School of Automation and Electrical Engineering, University of Science and Technology Beijing, Beijing 100083, China; ^3^School of Informatics, Linyi University, Linyi 276005, China; ^4^Traditional Chinese Internal Medicine Key Laboratory of China Education Ministry, Dongzhimen Hospital, Beijing University of Chinese Medicine, Beijing 100700, China

## Abstract

ChiCTR-TRC-11001263 study was the first large-scale double-blind randomized placebo-controlled traditional Chinese medicines (TCMs) and adefovir (ADV) antihepatitis B virus (HBV) infection trial in the world. A total of 560 hepatitis B e antigen- (HBeAg-) positive Chinese patients with chronical HBV were randomly classified, in 1 : 1 ratio, into two groups: experimental group (EXG) receiving TCMs + ADV and controlled group (CTG) receiving ADV + TCM-placebo treatment for 48 weeks. This paper introduces two models to model and simulate the evolutions of dynamics for the complete-response patients and the poor-response patients in EXG and CTG, respectively. The stimulated mean HBV DNA and alanine aminotransferase (ALT) levels were close to the patients' experimental data. Analysis and simulations suggest that the activated patients' immune functions by TCMs + ADV may not only clear infected hepatocytes, but also clear HBV, which made the complete-response patients' mean serum HBV DNA levels in EXG reduce rapidly 12 weeks' earlier than the ones in CTG. One can assume that both the TCMs and ADV have the function of preventing complete-response patients' infected hepatocytes from being injured by cytotoxic T lymphocytes (CTLs); the patients' activated immune cells may also block HBV replications.

## 1. Author Summary

Nucleoside analogues (NAs), such as lamivudine, adefovir, entecavir, and telbivudine, suppress HBV replication and result in the improvement of the liver architecture. Some TCMs are able to activate patients' immune function because patients' serum HBeAg levels may reduce rapidly much earlier before their serum HBV DNA levels decrease significantly. ChiCTR-TRC-11001263 was the first international registered ADV + TCM-placebo (control group CTG) and TCM + ADV-placebo switching to TCM + ADV (experimental group EXG) anti-HBV infection therapy trial. Based on Nowak et al.'s uninfected cell-infected cell-free virus basic virus infection model, this paper introduces two models with additional immune variable and alanine aminotransferase loads to describe and understand the two group patients' dynamics for anti-HBV infection therapy. The results include the determinations of the model parameters, predicting the outcome of the long-term treatment, finding that both the TCMs and ADV may have the function of preventing complete-response patients' infected hepatocytes from being injured by CTLs; activated CTLs may also play the role of blocking HBV replications; HBeAg seroconversion may be defined as a predictor that patients can keep their activated immune function via one-year additional treatment, then ending their therapy.

## 2. Introduction

Hepatitis B is a life-threatening liver infection caused by hepatitis B virus (HBV), which can cause chronic liver disease and make people die of cirrhosis of the liver and liver cancer. Two billion people worldwide have been infected with HBV and more than 400 million have chronic (long-term) liver infections. An estimated 1 million people die every year due to the consequences of hepatitis B [1].

The goal of anti-CHB infection treatment is to achieve sustained suppression of HBV DNA and remission of liver disease [2]. Nucleoside analogues, such as lamivudine, adefovir, entecavir, and telbivudine, are popular drugs to treat HBV infection. The main role of nucleoside analogues is to block the replication of HBV DNA in vivo.

Some TCMs anti-HBV infection therapies have the advantages of rare viral mutation, rare side, and cheap price. Lines of evidence show that TCMs can regulate CHB patients' immune functions [3].

Monotherapy may have low response rates. Most CHB patients need long-term medication, which can maintain a low response rate after withdrawing drugs and result in higher rate of drug resistance [4–8]. The disadvantages of some NA monotherapies limit the clinical application of CHB patients' treatments.

NA + TCM therapy has better efficacy than monotone treatment, which is able to increase proportion of patients' achieving HBeAg loss and clear HBV directly without damaging patients' hepatocytes [3, 9].

Modelling the dynamics of HBV infection and other virus infections has attracted considerable attentions. Mathematical models play a significant role in improving understanding of the dynamics of the HBV infections in vivo. The models typically used to study HBV dynamics in vivo tend to focus on healthy cells, free virus, and infected cells [10, 11].

The basic viral infection dynamic mathematical model (BVIM) proposed by Nowak et al. [12, 13] has been widely used in the study of the dynamic of infectious agents such as hepatitis B, C and HIV. The BVIM has the following form [12]:

(1) $$\begin{array}{c} \dot{x}=\lambda -dx-bvx,\\ \dot{y}=bvx-ay,\\ \dot{v}=ky-uv, \end{array}$$

where *x*, *y*, and *v* are the numbers of uninfected cells, virus-infected cells, and free virus, respectively. Uninfected cells are produced at a constant rate *λ*, die at rate *dx*, and become infected at rate *bvx*. Virus-infected cells are produced at rate *bvx* and die at rate *ay*. Free virus is produced from virus-infected cells at rate *ky* and is removed at rate *uv*.

Equation (1) has an infection free-steady state *Q*_1_:

(2) $$\begin{array}{c} Q_{1}=\left(\frac{\lambda }{d},0,0\right), \end{array}$$

representing an infected person's complete recovery. Equation (1) has also an endemic steady state *Q*_2_:

(3) $$\begin{array}{c} Q_{2}=\left(\frac{au}{\beta k},\frac{\lambda }{a}\left(1-\frac{1}{R_{0}}\right),\frac{d}{\beta }\left(R_{0}-1\right)\right),\;R_{0}=\frac{\lambda bk}{adu} \end{array}$$

representing an infected person's persistent infection. Here, *R*_0_ is called the basic virus reproductive number of model (1).

It has been proved that if *R*_0_ ≤ 1, then the infection-free steady state of the model (1) is globally attractive; otherwise the endemic steady state of the model (1) is globally attractive [14].

Since *λ*/*d* in *R*_0_ represents the total number of uninfected cells of the patient's target organ, this implies that an individual with a larger liver will be more difficult to be cured than a person with a smaller one. The meaning of *R*_0_ is questionable. Recently, some amended basic viral infection models (ABVIM) [10, 15, 16] are established. One of them takes the following form [15]:

(4) $$\begin{array}{c} \dot{x}=\lambda -dx-\frac{bvx}{x+y},\\ \dot{y}=\frac{bvx}{x+y}-ay,\\ \dot{v}=ky-uv, \end{array}$$

where the meanings of the variables *x*, *y*, and *v* and the parameters *λ*, *d*, *a*, *k*, and *u* are the same as those given in model (1). (*bvx*)/(*x* + *y*) is the viral infected rate of uninfected cells by free virus and produced rate of virus from virus-infected cells.

The ABVIM has a basic virus reproductive number *R*_0_ = *bk*/(*au*), which is independent on the total number of cells of the patient's target organ. It has been proved that if *R*_0_ ≤ 1, then the infection free steady state is globally attractive; otherwise the endemic steady state is globally attractive [15, 17].

During the process of viral infections, the immune response has been shown to be universal and necessary to eliminate or control the disease [18, 19]. Actually, in most virus infections, cytotoxic T lymphocytes (CTLs) play a critical role in antiviral defense by attacking virus-infected cells [20]. 

Therefore, many viral infection dynamic mathematical models with immune response have been studied in recent years [3, 13, 20–22]. One of them has the following form [13]:

(5) $$\begin{array}{c} \dot{x}=\lambda -dx-bvx,\\ \dot{y}=bvx-ay-k_{1}ye,\\ \dot{v}=ky-uv,\\ \dot{e}=k_{2}y-k_{3}e, \end{array}$$

where the meanings of the variables *x*, *y*, and *v* and the parameters *λ*, *d*, *b*, *a*, *k*, and *u* are the same as those given in model (1). The variable *e* represents the number of cytotoxic T lymphocytes (CTLs). CTLs are produced at rate *k*_2_ *y* and die at rate *k*_3_ *e*. The term *k*_1_ *ye* is the death rate of virus-infected cells caused by immune response. Model (5) has a basic virus reproductive number *R*_0_ = *λ* *bk*/(*adu*), which is also dependent on the total number of cells of the patient's target organ. The infection free-steady state *Q*_1_ = (*λ*/*d*, 0,0, 0) of model (5) is independent on the parameters *k*_2_ and *k*_3_ which relate to the production of CTLs.

Based on the experimental data and previous researches on the dynamics of virus infection model [3, 10, 12, 13, 15, 16, 20–22], this paper introduces two mathematical models to model, simulate, and analyze the dynamics of the evolutions of patients' mean serum HBV DNA and ALT levels and make long-term prediction for the complete-response patients and the poor-response patients for ADV monotherapy and TCM + ADV combination therapy.

## 3. Methods

### 3.1. Experiment

ChiCTR-TRC-11001263 study was a double-blind randomized placebo-controlled trial. ADV and two kinds of TCMs, Tiaoganjianpihuoxue grain (TCM1) and Tiaoganjieduhuashi grain (TCM2), were used in the trial. TCM1 consists of 13 herbal ingredients, and TCM2 consists of 15 herbal ingredients. A total of 560 Chinese HBeAg-positive CHB patients were randomly classified into, in 1 : 1 ratio, two groups: control group (CTG) and experimental group (EXG).

The patients' plasma HBV DNA level baselines were 3 log⁡_10_copies/mL ~  8 log⁡_10_⁡  copies/mL by PCR assay. And the alanine aminotransferase (ALT) level baselines were 2 ULN~12 ULN (Disease: ULN, upper limit of normal), where the abbreviation ULN represents “upper limit of normal”. Total bilirubin (TBIL) load baselines were less or equal to 3ULN.

The patients in CTG received ADV (10 mg, once daily) + TCM-placebo (twice daily) for 48 weeks. The patients in EXG were divided into 3 subgroups as follows.

- Group EXG1 has 207 patients whose ALT levels were larger than 2 ULN and less than 6 ULN.
- Group EXG2 has 39 patients whose ALT levels were larger than 6 ULN and less than 12 ULN.
- Group EXG3 has 34 patients whose HBeAg levels were less than 60 S/CO.

The patients' numbers of the corresponding three subgroups in CTG are 206, 35, and 39, respectively. The control group and the experimental three subgroups have the same characteristics (ITT).

The experimental schemes of the three sub-EXGs were designed as follows.

- The patients in EXG1 received TCM1 (twice daily) + ADV-placebo (10 mg once daily) for the first 24 weeks and then switched to TCM2 + AD for additional 24 weeks.
- The patients in EXG2 received TCM2 (twice daily) + ADV-placebo (10 mg once daily) for the first 24 weeks and then switched to receive TCM2 + AD for additional 24 weeks.
- The patients in EXG3 continuously received TCM2 (twice daily) + ADV (10 mg once daily) for 48 weeks.
- During the first 24-week therapy if a patient's ALT level in EXG2 was larger than 8 × ULN, then the patient switched to receive TCM2 + ADV-placebo until the 24th week and then switched to receive TCM2 + ADV for additional 24 weeks.
- For any one in EXG1 or EXG2, if a patient's HBeAg level was less than 60 S/CO, or ALT level was larger than 12 × ULN, or TBIL level was larger than 3 ×  ULN, then the patient switched to receive the scheme of EXG3 for therapy.

The main function of TCM1 is to regulate patients' immune abilities, and the main role of TCM2 is to block the repletion of HBV. However, efficacy of TCM2 is limited, and it needs NA (e.g., ADV) for combination therapy to increase its efficacy.

The therapy scheme suggests that the patients with lower immune abilities whose ALT levels were less than 6 ULN or HBeAg levels were larger than 60 S/Co or TBIL levels were less than 3 ULN should receive only TCM1 therapy to regulate their immune functions for the first 24 weeks if their tested items did not change to the levels given in item (e).

The conditions item (e) may be a criterion which makes corresponding patients switch to use TCM2 + ADC scheme for further therapy.

Consequently, the purpose of the above therapy scheme was to expect that the 24-week therapy would make some patients in groups EXG1 and EXG2 achieve the conditions in item (e). And then the patients in the three groups received the TCM2 + ADV combination treatment for additional 24 weeks.

Some virologic and biochemical responses of the six subgroups are listed in Table 1 (also see [9]). The results show that TCM + ADV anti-HBV combination therapy resulted in increased proportion of patients achieving HBeAg loss in the EXG1 and EXG3 versus the CTG1 and CTG3 at week 48. The other virologic and biochemical responses of the controlled group and the experimental group had not significant differences.

At week 48, there were 28 and 31 patients in EXG and CTG who achieved complete response (denoted by CEXG and CCTG), respectively. Meanwhile, there were 42 and 55 patients in EXG and CTG responded poorly (denoted by PEXG and PCTG). Here complete response is defined as HBV DNA level being lower than undetectable level (<1000 copies/mL) and HBeAg seroconversion (HBeAg < 1 and anti-HBe < 1); poor response is defined as less than 1 log_10_ copies/mL decrease in HBV DNA level from the baseline at the 48th week. 

**Figure 1:**
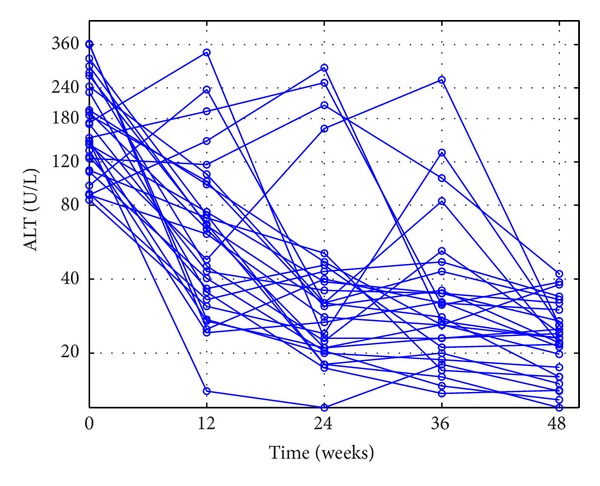
Outcomes of the complete-response patients' ALT loads in the experimental group during the 48-week therapy.

**Figure 2:**
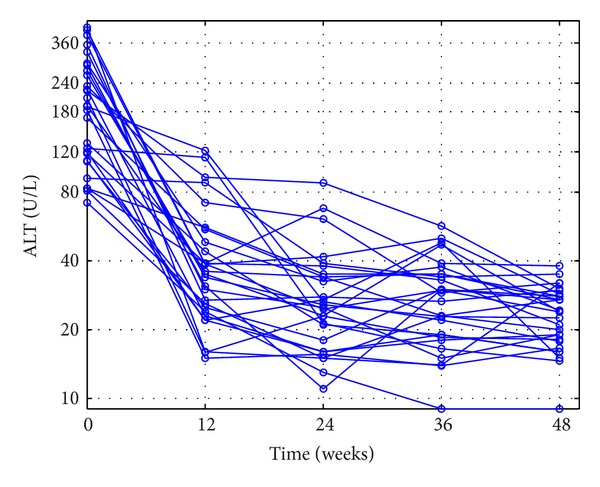
Outcomes of the complete-response patients' ALT loads in the control group during the 48-week therapy.

**Figure 3:**
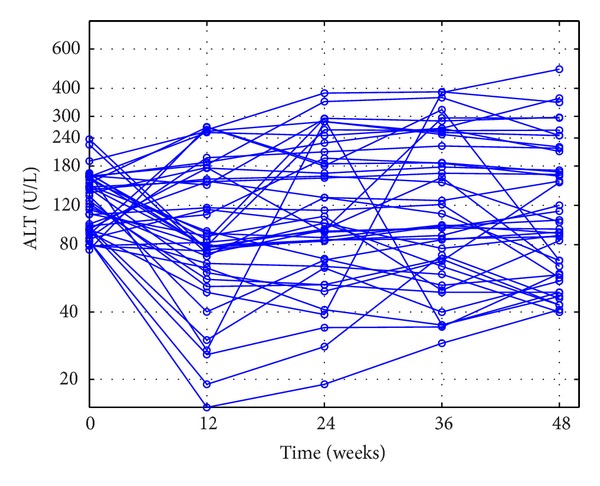
Outcomes of the poor-response patients' ALT loads in the experimental group during the 48-week therapy.

**Figure 4:**
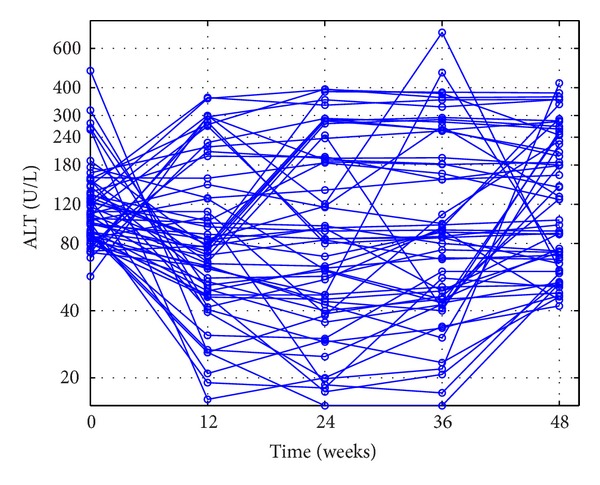
Outcomes of the poor-response patients' ALT loads in the control group during the 48-week therapy.

The outcomes of the patients' ALT loads in CEXG and CCTG are shown in Figures 1, 2, 3, and 4, respectively. The patients' mean HBV DNA levels, ALT loads, and HBeAg levels are listed in Table 2 (also see [23]).

Figures 1–4 and the data in Table 2 suggest that the main function of the TCMs is to regulate the patients' immune functions. The additional 24-week TCM + ADV therapy speeded up the patients' enhancement of immune functions. This observation motivates us to introduce two models to describe the dynamics of anti-HBV infection with ADV and TCMs + ADV in the next section.

### 3.2. Models

Based on the previous work [3, 10, 12, 13, 15, 16, 20–22] and the above analysis, we use model (6) to describe the dynamics of the CTG with the ADV anti-HBV infection therapy (similar to that proposed in [22] which does not include the ATL level variable *w*):

(6) $$\begin{array}{c} \dot{x}=\lambda -dx-\left(1-m\right)\frac{bvx}{x+y},\\ \dot{y}=\left(1-m\right)\frac{bvx}{x+y}-ay-\frac{k_{1}ye}{x+y},\\ \dot{v}=\left(1-n\right)ky-uv,\\ \dot{e}=k_{2}\left(x+y\right)-k_{3}e,\\ \dot{w}=k_{5}+k_{6}{\left(\frac{k_{1}ye}{x+y}\right)}^{3}-k_{7}w. \end{array}$$

By the similar reasons, we introduce model (7) to describe the dynamics of the EXG with the TCM + ADV anti-HBV infection therapy (similar to [3, 24]):

(7) $$\begin{array}{c} \dot{x}=\lambda -dx-\left(1-m\right)\frac{bvx}{x+y},\\ \dot{y}=\left(1-m\right)\frac{bvx}{x+y}-ay-\frac{k_{1}ye}{x+y},\\ \dot{v}=\left(1-n\right)ky-uv-\frac{k_{4}ve}{x+y},\\ \dot{e}=k_{2}\left(x+y\right)-k_{3}e,\\ \dot{w}=k_{5}+k_{6}{\left(\frac{k_{1}ye}{x+y}\right)}^{3}-k_{7}w. \end{array}$$

Here, the meanings of the variables *x*, *y*, *v*, and *e* and the parameters *λ*, *d*, *b*, *a*, *k*, and *u* are the same as those given in model (1); *e* represents the number of CTLs which are produced at rate *k*_2_(*x* + *y*) and die at rate *k*_3_ *e*; (*k*_1_ *ye*)/(*x* + *y*) is the death rate of virus-infected cells generated by immune killing; (*k*_4_ *ve*)/(*x* + *y*) is the clearing rate of virus generated by some specific immune abilities activated via antivirus infection therapy. The variable *w* represents the serum ALT levels. A liver without immune attacking produces ALT at rate *k*_5_, and ALT dies at rate *k*_7_ *w*. A CHB patient's liver produces ALT at rate *k*_5_ + *k*_6_(*k*_1_ *ye*/(*x* + *y*))^3^. *m*, *n* (0 < *m*, *n* < 100%) are the treatment efficacy variables during the anti-HBV treatment.

Model (6) and model (7) both have the same infection-free equilibrium *Q*_1_:

(8) $$\begin{array}{c} Q_{1}=\left(\frac{\lambda }{d},0,0,\frac{k_{2}\lambda }{k_{3}d},\frac{k_{5}}{k_{7}}\right). \end{array}$$

Model (6) has an endemic equilibrium *Q*_2_^1^:

(9) $$\begin{array}{c} Q_{2}^{1}=\left(\overset{-}{x},\overset{-}{y},\overset{-}{v},\overset{-}{e},\overset{-}{w}\right), \end{array}$$

representing persistent virus infection, where

(10) $$\begin{array}{c} \overset{-}{x}=\frac{\lambda u\left(1+c_{1}\right)}{du\left(1+c_{1}\right)+bkc_{1}},\;\;\overset{-}{y}=c_{1}\overset{-}{x},\\ \overset{-}{v}=\frac{kc_{1}\overset{-}{x}}{u},\;\;\overset{-}{e}=\frac{k_{2}\overset{-}{x}\left(1+c_{1}\right)}{k_{3}},\\ \overset{-}{w}=\frac{1}{k_{3}k_{7}}\left(k_{3}k_{5}+kk_{2}k_{6}c_{1}\overset{-}{x}\right),\\ c_{1}=\frac{R_{0}-1-c_{2}}{1+c_{2}},\;\;R_{0}=\frac{bk}{au},\;\;c_{2}=\frac{k_{1}k_{2}}{ak_{3}}. \end{array}$$

Model (7) has an endemic equilibrium *Q*_2_^2^:

(11) $$\begin{array}{c} Q_{2}^{2}=\left(\overset{-}{x},\overset{-}{y},\overset{-}{v},\overset{-}{e},\overset{-}{w}\right), \end{array}$$

where

(12) $$\begin{array}{c} \overset{-}{x}=\frac{\lambda }{d+\left(bc_{1}c_{2}\right)/1+c_{2}},\;\overset{-}{y}=c_{2}\overset{-}{x},\\ \overset{-}{v}=c_{1}c_{2}\overset{-}{x},\;\;\overset{-}{e}=\frac{k_{2}\left(1+c_{2}\right)\overset{-}{x}}{k_{3}},\\ \overset{-}{w}=\frac{k_{5}\left(\overset{-}{x}+\overset{-}{y}\right)+k_{1}k_{6}\overset{-}{y}\;\overset{-}{e}}{k_{7}\left(\overset{-}{x}+\overset{-}{y}\right)},\\ c_{1}=\frac{kk_{3}}{uk_{3}+k_{2}k_{4}},\;\;c_{2}=\frac{bc_{1}k_{3}-ak_{3}-k_{1}k_{2}}{ak_{3}+k_{1}k_{2}}. \end{array}$$

For model (6), the basic virus reproductive number is

(13) $$\begin{array}{c} R_{0}=\frac{kb\left(1-m\right)\left(1-n\right)}{au\left(1+\left(k_{1}k_{2}/ak_{3}\right)\right)}. \end{array}$$

For model (7), the basic virus reproductive number is

(14) $$\begin{array}{c} R_{*}=\frac{kb\left(1-m\right)\left(1-n\right)}{au\left(1+\left(k_{1}k_{2}/ak_{3}\right)\right)\left(1+\left(k_{2}k_{4}/uk_{3}\right)\right)}. \end{array}$$

Similar to [22], we can prove the following theorems.

**Theorem 1**

Let *R*_0_ be defined by (13). If *R*_0_ < 1, then the infection-free equilibrium *Q*_1_ of (6) is locally stable.

**Theorem 2**

If

(15) $$\begin{array}{c} \frac{kb\left(1-m\right)\left(1-n\right)}{au}<1, \end{array}$$

then the infection-free equilibrium *Q*_1_ of (6) is globally asymptotically stable.

Similar to [3, 24], we can prove the following theorems.

**Theorem 3**

Let *R*_∗_ be defined by (14). If *R*_∗_ < 1, then the infection-free equilibrium *Q*_1_ of (7) is locally stable.

**Theorem 4**

If

(16) $$\begin{array}{c} \frac{kb\left(1-m\right)\left(1-n\right)}{au}<1, \end{array}$$

then the infection free equilibrium *Q*_2_ of (7) is globally asymptotically stable.

## 4. Numerical Simulation

In this subsection, in order to interpret more clearly the specific role of TCM in the anti-HBV infection therapy, we use model (7) and model (6) to simulate the dynamics of the evolutions of mean serum HBV DNA and ALT levels and make long-term prediction for the CRP and PRP in CTG and EXG anti-HBV infection therapies, respectively.

### 4.1. Simulations for Complete-Response Patients' Dynamics in EXG

Model (7) is used to simulate complete-response patients' dynamics for TCM + ADV anti-HBV infection therapy. Use the methods in [3, 15, 17, 22] to determine approximately the parameters in model (7) as follows.

(1) Because a human liver contains about 2 × 10^11^ hepatocytes [13], we obtain

(17) $$\begin{array}{c} \frac{\lambda }{d}\approx 2\times 10^{11}. \end{array}$$

(2) Since the half-life of a hepatocyte is about half a year [25], we get

(18) $$\begin{array}{c} d=-\frac{\ln\left(0.5\right)}{183}. \end{array}$$

(3) Assuming the natural death rate of infected hepatocytes is the same as that of uninfected hepatocytes, hence we obtain

(19) $$\begin{array}{c} a=d. \end{array}$$

(4) A CHB patient typically has between *δ* = 5% and *δ* = 40% infected hepatocytes [13]. Different CHB patient's serum HBV DNA load varies ranging from $\overset{-}{v}=10^{3}$ cps/mL to $\overset{-}{v}=10^{12}$ cps/mL. Hence, we assume that *δ* and $\overset{-}{v}$ have the following relation:

(20) $$\begin{array}{c} \delta =p+q\overset{-}{v}. \end{array}$$

We can calculate *p* and *q* via the following equations:

(21) $$\begin{array}{c} 5\%=p+q\times 10^{3},\\ 40\%=p+q\times 10^{12}. \end{array}$$

Consequently, we obtain

(22) $$\begin{array}{c} \delta =0.05+3.5\times 10^{-13}\overset{-}{v}. \end{array}$$

(5) In the complete-response patients in EXG for TCM + ADV anti-HBV infection therapy, $\overset{-}{v}=4.4556\;\times \;10^{7}$ cps/mL. Hence, we calculate

(23) $$\begin{array}{c} \delta \approx 0.050016. \end{array}$$

(6) Chronic HBV infection makes some infected hepatocytes undergo apoptosis and be replaced by hepatic stellate cells [26]. Define a parameter *δ*_0_, and the patient's hepatocytes are reduced by (1 − *δ*_0_) × 100 percent. Hence, we get

(24) $$\begin{array}{c} \overset{-}{x}+\overset{-}{y}=\delta_{0}\frac{\lambda }{d}, \end{array}$$

where we choose *δ*_0_ = 0.95. Furthermore, we obtain

(25) $$\begin{array}{c} \overset{-}{x}=\left(1-\delta \right)\delta_{0}\frac{\lambda }{d},\\ \overset{-}{y}=\delta \delta_{0}\frac{\lambda }{d}. \end{array}$$

(7) Assuming the half-life of a virus is about one day [13], we obtain

(26) $$\begin{array}{c} u=0.67. \end{array}$$

(8) Assuming that the baseline *k*_4_ = 0, we get

(27) $$\begin{array}{c} k=\frac{u\overset{-}{v}}{\overset{-}{y}}. \end{array}$$

(9) Since the half-life of CTLs is about 77 days [27], we obtain

(28) $$\begin{array}{c} k_{3}=-\frac{\ln\left(0.5\right)}{77}. \end{array}$$

(10) Because the half-life of ALT is about 2~3 days [28], we select

(29) $$\begin{array}{c} k_{7}=-\frac{\ln\left(0.5\right)}{2.5}. \end{array}$$

(11) We assume that 22 U/L is the mean normal ALT level because the complete response patients' mean ALT level was about 24 U/L at the week 48. When a human is healthy, *k*_1_ = 0 in model (7). Hence, we can obtain that

(30) $$\begin{array}{c} k_{5}=22\times 3\times k_{7} \end{array}$$

 because an individual has about about 3-liter serum. 

(12) Solving the equilibrium point equation gives

(31) b=λ−dx−v−(1−δ),  k1=bv−(1−δ)−ay−δe−,k2=k3e−dδ0λ,  k6=(k7w−−k5)(x−+y−)k1y− e−.

(13) Because a healthy Chinese has about 600 ± 300 counts/*μ*L CD8 + T cells, we assume that

(32) $$\begin{array}{c} e_{0}=200\times 3\times 10^{6}. \end{array}$$

(14) Select the mean serum HBV DNA level 4.4556 × 10^7^ copies/mL at week 0 as the initial value, and an individual have 3-liter serum; hence we determine

(33) $$\begin{array}{c} v_{0}=4.4556\times 10^{7}\times 3\times 10^{3}. \end{array}$$

(15) Select the mean serum ALT level value 180.06 U/L (see Table 2) at week 0 as the initial value, and an individual have 3-liter serum; hence we obtain

(34) $$\begin{array}{c} w_{0}=180.06\times 3. \end{array}$$

(16) Select *m* = 0 since none of the available nucleoside analogues inhibitors have been shown to prevent infection of uninfected hepatocytes [29]. 

In order to agree with the experimental data, the parameters *n*, *k*, *k*_1_, *k*_2_, and *k*_4_ need to be changed during the treatment. Their values are listed in Table 3.

Selecting the following initial condition

(35) (x0,y0,v0,e0,w0)   =((1−δ)δ0λd,δδ0λd,4.4556  ×107×3×103,   200×3×106,180.06×3),

then one can simulate the dynamics of the complete-response group in EXG for TCM + ADV anti-HBV infection therapy.

### 4.2. Simulations for Complete-Response Patients' Dynamics in CTG

Model (6) is used to simulate the complete-response patients' dynamics in CTG for ADV anti-HBV infection therapy. The parameters *λ*/*d*, *d*, *a*, *δ*_0_, *u*, *k*, *b*, *k*_1_, *k*_2_, *k*_3_, *k*_5_, *k*_6_, and *k*_7_ in model (6) have the same values as those given in (1)~(3) and (6)~(13) in the above section. Consider the following.

1. Substituting $\overset{-}{v}=5.8337\times 10^{7}$ cps/mL into formula (22) gives *δ* = 0.05002.
2. Since the mean serum HBV DNA load value at week 0 is 5.8337 × 10^7^, it follows that *v*_0_ = 5.8337 × 10^7^ × 3 × 10^3^ via (14) in the above section.
3. Since the mean serum ALT level value at week 0 is 198.02 U/L (see Table 2), it follows that *w*_0_ = 198.02 × 3 via (15) in the above section.

The parameters *n*, *k*_1_, and *k*_2_ have changed during the treatment. Their values are listed in Table 4.

 Selecting the following initial condition:

(36) (x0,y0,v0,e0,w0) =((1−δ)δ0λd,δδ0λd,5.8337×107×3×103,   200×3×106,198.02×3),

then one can simulate the complete-response patients' dynamics in CTG.

### 4.3. Simulations for Poor-Response Patients' Dynamics in EXG

Model (7) is used to simulate the poor-response patients' dynamics of in EXG for TCM + ADV anti-HBV infection therapy. The parameters *λ*/*d*, *d*, *a*, *δ*_0_, *u*, *k*, *b*, *k*_1_, *k*_2_, *k*_3_, *k*_4_, *k*_5_, *k*_6_, and  *k*_7_ in model (7) have the same values as those given in (1)~(3) and (6)~(13) in the above section. Consider the following.

1. Substituting $\overset{-}{v}=2.6685\;\times 10^{8}$ cps/mL into formula (22) gives *δ* = 0.050093.
2. Since the mean serum HBV DNA load value at week 0 is 2.6685 × 10^8^, it follows that *v*_0_ = 2.6685 × 10^8^ × 3 × 10^3^.
3. Since the mean serum ALT level value at week 0 is 127.47 U/L, it follows that *w*_0_ = 127.47 × 3.

The parameters *n*, *k*, *k*_2_, and *k*_4_ have changed during the treatment. Their values are listed in Table 5.

Selecting the following initial condition:

(37) (x0,y0,v0,e0,w0) =((1−δ)δ0λd,δδ0λd,2.6685×108×3×103,   200×3×106,127.47×3),

then one can simulate the poor-response patients' dynamics in EXG for TCM + ADV anti-HBV infection therapy.

### 4.4. Simulations for Poor-Response Patients' Dynamics in CTG

Model (6) is used to simulate the poor-response patients' dynamics in CTG for ADV anti-HBV infection therapy. The parameters *λ*/*d*, *d*, *a*, *δ*_0_, *u*, *k*, *b*, *k*_1_, *k*_2_, *k*_3_, *k*_5_, *k*_6_, and *k*_7_ in model (6) have the same values as those given in (1)~(3) and (6)~(13) in the above section. Consider the following.

1. Substituting $\overset{-}{v}=1.8504\;\times \;10^{8}$ cps/mL into formula (22) gives *δ* = 0.050065.
2. Since the mean serum HBV DNA level at week 0 is 1.8504 × 10^8^, it follows that *v*_0_ = 1.8504 × 10^8^ × 3 × 10^3^ via (14) in the above section.
3. Since the serum ALT level value at week 0 is 128.36 U/L, it follows that *w*_0_ = 128.36 × 3.

The parameters *m*, *n*, *k*, *k*_1_, and *k*_2_ have changed during the treatment. Their values are listed in Table 6.

Selecting the following initial condition:

(38) (x0,y0,v0,e0,w0) =((1−δ)δ0λd,δδ0λd,1.8504×108×3×103,   200×3×106,128.36×3),

then one can simulate the dynamics of the poor response patients in CTG for ADV anti-HBV infection therapy.

## 5. Results

**Figure 5:**
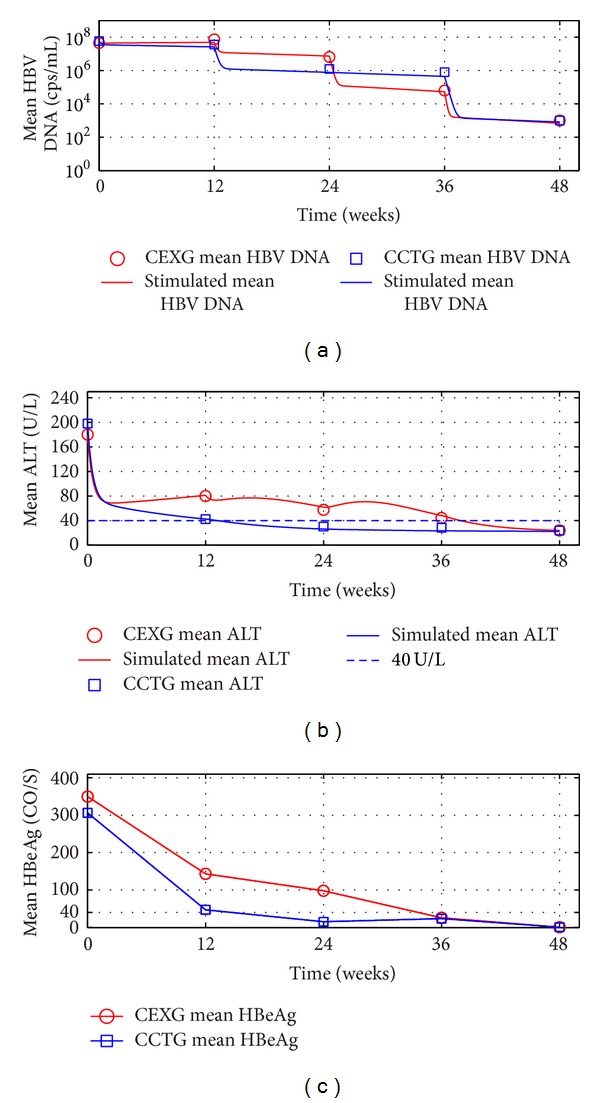
The outcomes of the complete-response patients' therapy efficacy in EXG and CTG. For 104-week treatments and 136-week followup: mean serum HBV DNA, ALT, and HBeAg levels. Solid lines: simulations of models (6) and (7). Circles and squares: complete-response patients' mean value experimental data in EXG and CTG, respectively. (a) Mean HBV DNA levels. (b) Mean ALT levels.

**Figure 6:**
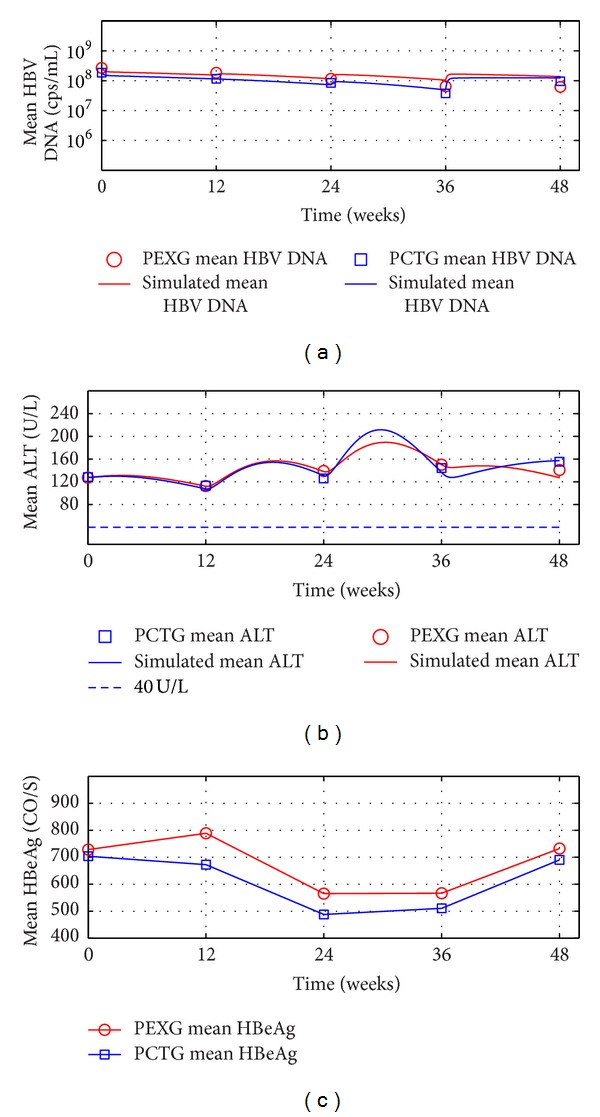
Outcomes of the complete-response patients' therapy efficacy in EXG and CTG. Mean serum HBV DNA, ALT, and HBeAg levels. Solid lines: simulations of models (6) and (7). Circles and squares: complete-response patients' mean value experimental data in EXG and CTG, respectively. (a) Mean HBV DNA levels. (b) Mean ALT levels. (c) Mean HBeAg levels.

The numerical simulations of the evolution dynamics of patients mean serum HBV DNA and ALT levels for the four subgroups are shown in Figures 5 and 6, respectively. Observe that the stimulated evolutions of the mean serum HBV DNA levels and ALT levels are close to the experimental data.

The numerical simulations of the patients' dynamics of the anti-HBV infection therapies give the following results.

(1) At the week 0 (baseline), the basic virus reproductive numbers *R*_∗_′*s* and *R*_0_′*s* of the 4 subgroups CEXG, CCTG, PEXG, and CTG are 1.0526, 1.0527, 1.0527, and 1.0527, respectively. This can interpret why they become virus-persistent CHB patients.

After the 48-week therapy, the basic virus reproductive numbers *R*_∗_ and *R*_0_ of the two subgroups CEXG and CCTG were reduced to 1.1 × 10^−4^ and 7.9 × 10^−5^, respectively. Further simulations show that it needs about 6.5 and 6.8 years of treatment to make all infected hepatocytes be replaced by normal ones, respectively.

After the 48-week therapy, the basic virus reproductive numbers *R*_∗_ and *R*_0_ of the two subgroups PEXG and PCTG were only reduced to 0.84085 and 0.87121, respectively. Further simulations show that the poor-response patients in EXG and CTG cannot be recovered completely until 20 years of treatments.

(2)Figure 5 and Table 2 show that the complete-response patients' mean serum HBV DNA levels in CEXG have reduced rapidly during week 24 and week 36, which was 12 weeks earlier than the complete response patients in CCTG.

In order to model this phenomenon, the parameter *k*_2_ in model (7) related to the production rate of CTLs has increased from 2*k*_2_ to 4*k*_2_ while the parameter *k*_4_ in model (7) has been designed to increase form 4*k*_1_ to 20*k*_1_ during week 24 and week 36. *k*_4_ represents the clearing rate of virus generated by some specific immune abilities activated via anti-HBV infection therapy, which can clear HBV directly.

Combining the trial data listed in Tables 1 and 2 and the modeling data given in Tables 3 and 4 follows that the TCMs + ADV combination therapy may offer superior efficacy for suppressing HBV replications than monotone ADV therapy.

(3) Comparing the model parameters given in Tables 3–6 gives the following.

- At week 48, the poor-response patients' parameter value on *n* is much smaller than the complete-response patients' one.
- During weeks 25 to 48, the poor-response patients' parameter value on *k* increased while the complete-response patients' one kept unchanged.

The above results imply that for the poor-response patients, the drug resistance made the therapy efficacy (parameter *n*) reduce rapidly, and the virus replication rate (parameter *k*_1_) was increased quickly.

## 6. Discussion

Based on the experimental data of CHB patients' serum HBV DNA levels and ALT levels, this paper introduces two differential equation models (6) and (7) to describe the CHB patients' dynamics for the ADV monotone treatment and the TCMs + ADV combination therapy. An amended term *k*_6_(*k*_1_ *ye*/(*x* + *y*))^3^ related to the ability for killing infected hepatocytes is included in the models to describe the evolution of the patients' ALT levels.

Making some simplified assumptions, one can determine 11 of the 13 parameters in (6) and (7). The simulation results are close to the patients' mean HBV DNA levels and mean ALT levels.

Based on the experimental data (see Table 2) and the simulation results, one can propose the following hypotheses.

*Hypothesis (a)*. Both the TCMs and ADV have the function that prevents complete-response patients' infected hepatocytes from being injured by CTLs; that is, the killing parameter *k*_1_′*s* in (6) and (7) becomes smaller than its baseline values.

This hypothesis may interpret why the complete-response patients' ALT loads decreased quickly while their HBV DNA levels decreased slowly or increased during the first 12-week therapy (see Tables 2, 3, and 4 and Figure 5).

Clinically, some patients with NA or TCM treatments may show serum HBV DNA levels to rebound higher than their baseline levels after cessation treatments. Hypothesis (a) may interpret that the patients kept the function of preventing infected hepatocytes from being injured by CTLs after stopping therapy.

The experimental data (see Table 2) and the simulation results (see Figure 6) suggest that the Chinese patients with high baseline HBV DNA levels, as well as HBeAg loads, and low baseline ALT levels may not obtain complete responses for the ADV or the CTMs + ADV treatments in 48 weeks.

The numerical simulations show that for the complete-response patients in EXG and CTG, it needs about 6.5 and 6.8 years of treatment to make all infected hepatocytes be replaced by normal if no virus mutations will appear and the efficacy of the therapy will be kept.

Clinically, a complete response CHB patient with nucleoside analogues treatment usually needs much longer times to obtain hepatitis B surface antigen loss. This fact suggests that patients' activated immune abilities may decrease as patients' HBV DNA levels decrease to very low levels.

At week 48, the very high efficacy (see *n* given in Tables 3 and 4 and Figure 5) of suppressing HBV replications makes us propose the second hypothesis.

*Hypothesis (b).* The efficacy of blocking HBV replications is not generated via TCMs and/or ADV alone. The CTLs (represented by variable *e* in (6) and (7)) efficiently control HBV replication by noncytolytic mechanisms [30] contributing also to block HBV replications.

This hypothesis may interpret why some patients' HBV DNA levels reduced rapidly at some specific time during their treatments because the activated noncytolytic mechanisms of CTL may play roles.

Based on a review article on the endpoints of hepatitis B treatment [31] and hypothesis (b), one can propose the following.

*Hypothesis (c)*. For complete-response CHB patients with nucleoside analogues treatments, additional one-year consolidation therapy can make most patients keep their activated immune abilities (parameter *k*_2_ in (6) and (7)), contributing to the treatment efficacy (parameter *n*) after finishing the consolidation treatment.

This hypothesis may interpret Recommendation 9 given in the Chronic Hepatitis B Guideline of the Asian-Pacific Association for the Study of the Liver.

For oral antiviral agents, in HBeAg-positive patients, treatment can be stopped when HBeAg seroconversion with undetectable HBV-DNA has been documented on 2 separate occasions at least 6 months apart [32].

**Figure 7:**
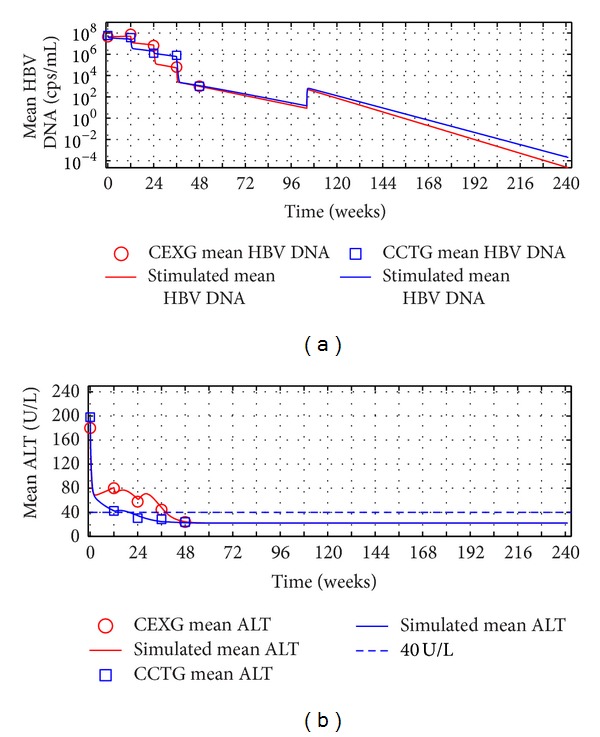
Outcomes of the poor-response patients' therapy efficacy in EXG and CTG. Mean serum HBV DNA, ALT, and HBeAg levels. Solid lines: simulations of models (6) and (7). Circles and squares: poor-response patients' mean value experimental data in EXG and CTG, respectively. (a) Mean HBV DNA levels. (b) Mean ALT levels. (c) Mean HBeAg levels.

Based on hypothesis (c), we assume that after finishing two-year treatment, the complete-response patients in EXG and CTG keep their immune parameter *k*_2_ unchanged, *k*_1_ returns to baseline, *k*_4_ = 0, and efficacy parameter *n* reduces to 0.99. The simulated evolutions dynamics of HBV DNA levels and ALT levels are shown in Figure 7. Observe that the treatment benefits are kept.

Modeling the dynamics for the ADV monotone treatment and the TCMs + ADV combination therapy may also provide some theoretical interpretation for the medical statistic results (see Table 1).

Since the TCMs + ADV therapy made the patients have an additional immune term (see the third equation in (7)), the TCMs + ADV therapy significantly resulted in increased proportion of the patients achieving HBeAg loss in the experimental group (see Table 1).

The modeling analysis with the experimental data analysis motivates to propose the previous three hypotheses, which may interpret some clinical experience judgements. The dynamics of anti-HBV infection therapy are very complex. It is difficult to set up mathematical model to describe them accurately. However, modelling dynamics of anti-HBV infection therapy would enable a better understanding, prediction, and design of anti-HBV infection treatments.

## Figures and Tables

**Table 1:** Virologic and biochemical responses at week 48.

Group	HBeAg loss	DNA <10^3^	ALT < 1 ULN
EXG1 *N* (%)	47/207 (22.71%)	62/207 (29.95%)	118/207 (57%)
CTG1 *N* (%)	26/206 (12.62%)	55/205 (26.83%)	121/206 (58.74)
*P* value	0.0106	0.5529	0.7972
EXG2 *N* (%)	15/39 (38.46%)	21/39 (53.85%)	28/39 (71.70%)
CTG2 *N* (%)	11/35(31.43%)	20/35 (57.14%)	24/35 (68.57%)
*P* value	0.6974	0.9596	0.9616
EXG3 *N* (%)	21/34 (61.76%)	17/34 (50.00%)	24/34 (70.59%)
CTG3 *N* (%)	13/39 (33.33%)	23/39 (58.97%)	29/39 (74.36%)
*P* value	0.0282	0.5942	0.9225

**Table 2:** Mean HBV DNA levels, ALT levels, and HBeAg levels at different weeks.

Group	Item	Weeks				
		0	12	24	36	48
CEXG	DNA	4.46*e* + 7	7.41*e* + 7	6.49*e* + 6	63004	<1000
CCTG	DNA	5.83*e* + 7	3.62*e* + 7	1.25*e* + 6	7.97*e* + 5	<1000
CEXG	ALT	180.06	79.918	57.518	44.221	23.939
CCTG	ALT	198.02	42.329	30.235	28.439	23.784
CEXG	HBeAg	349.95	143.1	97.988	25.741	0.53036
CCTG	HBeAg	306.35	46.831	15.215	23.508	0.45258
PEXG	DNA	2.67*e* + 8	1.83*e* + 8	1.15*e* + 8	6.47*e* + 7	6.14*e* + 7
PCTG	DNA	1.85*e* + 8	1.15*e* + 8	8.37*e* + 7	3.72*e* + 7	9.59*e* + 7
PEXG	ALT	127.47	112.55	139.13	150.21	140.82
PCTG	ALT	128.36	113.18	126.19	144.36	155.41
PEXG	HBeAg	728.45	788.87	565.44	566.79	732.34
PCTG	HBeAg	703.73	672.5	488.11	510.91	690.28

**Table 3:** Parameter values in different weeks for the complete-response patients' model in EXG.

Weeks	*n*	*k*	*k*_1_	*k*_2_	*k*_4_
0~12	0.00	1.1*k*	0.65*k*_1_	*k*_2_	*k*_1_
13~24	0.75	*k*	0.60*k*_1_	1.5*k*_2_	4*k*_1_
25~36	0.995	*k*	0.60*k*_1_	3*k*_2_	20*k*_1_
37~48	0.9998	*k*	0.60*k*_1_	3*k*_2_	20*k*_1_

**Table 4:** Parameter values in different weeks for the complete-response patients' model in CTG.

Weeks	*n*	*k*_1_	*k*_2_
0~12	0.40	0.55*k*_1_	*k*_2_
13~24	0.95	0.55*k*_1_	1.5*k*_2_
25~36	0.95	0.55*k*_1_	2*k*_2_
37~48	0.9999	0.55*k*_1_	3*k*_2_

**Table 5:** Parameter values in different weeks for the poor-response patients' model in EXG.

Weeks	*n*	*k*	*k*_1_	*k*_2_	*k*_4_
0~12	0.20	*k*	0.90*k*_1_	1.4*k*_2_	2*k*_1_
13~24	0.30	*k*	0.80*k*_1_	2.4*k*_2_	2*k*_1_
25~36	0.44	1.2*k*	*k*_1_	4.5*k*_2_	2*k*_1_
37~48	0.18	3*k*	*k*_1_	4.0*k*_2_	9*k*_1_

**Table 6:** Parameter values in different weeks for the poor-response patients' model in CTG.

Weeks	*n*	*k*	*k*_1_	*k*_2_
0~12	0.20	*k*	0.9*k*_1_	1.4*k*_2_
13~24	0.30	*k*	0.8*k*_1_	2.4*k*_2_
25~36	0.44	1.3*k*	*k*_1_	4.5*k*_2_
37~48	0.40	3.5*k*	*k*_1_	4.0*k*_2_
